# Unique Presentation of Intra-Abdominal Testis: Small Bowel Obstruction

**DOI:** 10.5402/2011/579153

**Published:** 2011-06-14

**Authors:** Ibrahim E. Bassiouny, Tariq O. Abbas, Amani N. Alansari, Mansour A. Ali

**Affiliations:** Department of Pediatric Surgery, Hamad General Hospital, P.O. Box 3050, Doha, Qatar

## Abstract

We describe here a two-year-old male who required urgent laparotomy to relieve a strangulated small bowel caused by internal herniation around an intra-abdominal testis. This clinical presentation has not been reported previously.

## 1. Case Report

A 2-year-old boy presented with a 3-day history of diffuse abdominal cramping, nausea, vomiting, constipation, and progressive abdominal distension. There was no perinatal history of exposure to toxins, and prenatal ultrasound revealed no abnormalities.

**Figure 1:**
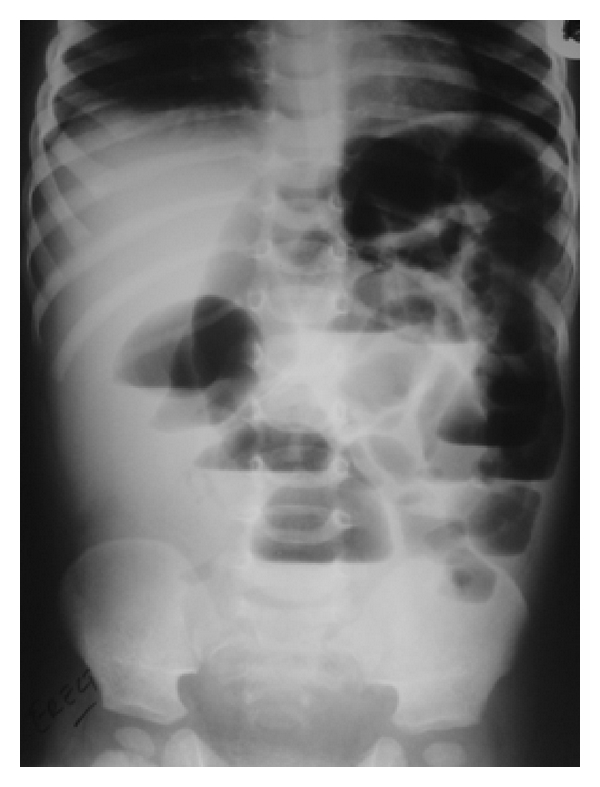
A plain abdominal X-ray shows small bowel intestinal obstruction.

Physical examination showed that the child was dehydrated, with a diffusely distended and tender abdomen, but no erythema or crepitus. His right scrotum was empty, with no testis present in his groin, while the contralateral testis was normal. Plain abdominal radiology revealed typical features of low-mechanical small bowel obstruction with numerous gas-fluid levels (Figure 1). Abdominopelvic sonography showed a fair amount of ascites in the pelvis with localized thickening of bowel loop walls in the right lower abdominal quadrant. Moreover, radiology showed no testis in his pelvis or scrotum. 

**Figure 2:**
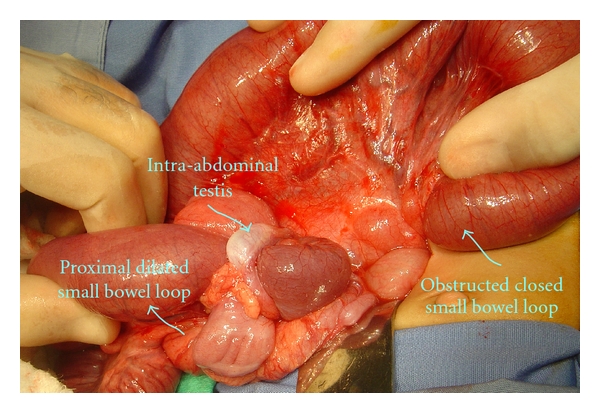
Herniating loop of terminal ileum in internal hernia, formed by adhesion of the gubernaculum of the right intra-abdominal testis to the terminal small bowel loop.

**Figure 3:**
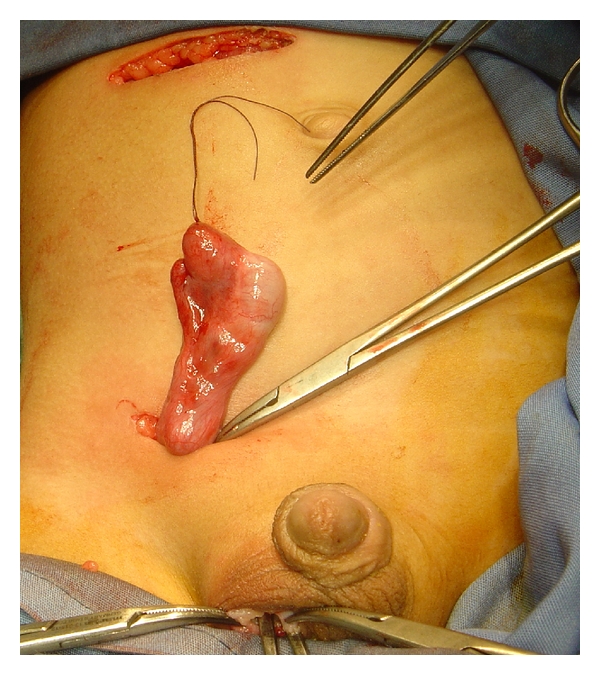
Relocation of the mobilized right testis to the scrotum via a small inguinal crease incision.

We performed urgent laparotomy through a right transverse supraumbilical incision that revealed an ischemic loop of terminal small bowel herniating below an intra-abdominal testis residing freely in the peritoneal cavity, with its gubernaculum adherent to the terminal ileum (Figure 2). We performed simple reduction of the ischemic loop, which regained its viability shortly afterward. The gubernaculum was freed from its attachment to the terminal ileum. The testis was viable and of normal size and could be brought down and fixed to the scrotum utilizing additional small inguinal creases and scrotal incisions (Figure 3). 

Postoperatively, the patient recovered from surgery without incident and was discharged from hospital after 3 days. Followup in our outpatient clinic 3 months later revealed a normal-sized right testis properly residing in the scrotum.

## 2. Discussion

Failure of a testis to reside properly within the scrotum after birth usually leads to increased rates of infertility and malignancy. Other dangers include increased risks of torsion and trauma, in addition to the psychological impact of an absent testis [1, 2]. 

Intestinal obstruction due to internal herniation is rare, reported in only 1% of patients with these obstructions [3]. Intestinal obstructions usually occur in the paraduodenal area (50%), but they may also occur in the transmesenteric, pericecal, intersigmoid, and supravesical areas [4]. Acute small bowel obstruction in the absence of previous surgery or external hernia is suggestive of internal hernia, despite its rarity. Compressed internal hernias may also cause strangulation and immediate gangrene [5].

We report what is, to our knowledge, the first case of a young child with an acutely strangulated small bowel that internally herniated around an intra-abdominal testis and was obstructed by it. It remains unclear whether the testis is an intra- or extraperitoneal organ. Although adult anatomy texts favor an extraperitoneal position, intraperitoneal testes have been observed in patients with gastroschisis, spigelian hernia, and testicular torsion [6]. 

Moreover, it has been suggested that, instead of following the processus vaginalis, the testis actually pulls out the peritoneal pouch into the scrotum [7, 8]. In contrast, the processus vaginalis may be an invagination of the peritoneal cavity into the extraperitoneal gubernacular mesenchyme, suggesting that the testes are extraperitoneal [9]. The findings in our patient, however, support the theory that the testes are intraperitoneal organs. 

## References

[1] Huff DS, Fenig DM, Canning DA, Carr MC, Zderic SA, Snyder HM (2001). Abnormal germ cell development in cryptorchidism. *Hormone Research*.

[2] Huff DS, Hadziselimović F, Snyder HM, Blyth B, Duckett JW (1991). Early postnatal testicular maldevelopment in cryptorchidism. *Journal of Urology*.

[3] Moran JM, Salas J, Sanjuán S (2004). Paramesocolic hernias: consequences of delayed diagnosis. Report of three new cases. *Journal of Pediatric Surgery*.

[4] Newsom BD, Kukora JS (1986). Congenital and acquired internal hernias: unusual causes of small bowel obstruction. *American Journal of Surgery*.

[5] Angood P, Gingalewski CA, Andersen DK, Townsend C Surgical complications. *Sabiston Textbook of Surgery: The Biological Basis of Modern Surgical Practice*.

[6] Pham SBT, Hong MKH, Teague JA, Hutson JM (2005). Is the testis intraperitoneal?. *Pediatric Surgery International*.

[7] Gingalewski CA, Grosfeld J, O’Neill J, Fonkalsrud E (2006). Other causes of intestinal obstruction. *Pediatric Surgery*.

[8] Adams JT, Schwartz SI, Shires GT, Spencer FC, Husser WC (1989). Abdominal wall, omentum, mesentery, retroperitoneum. *Principles of Surgery*.

[9] Backhouse KM (1964). The gubernaculum testis hunteri: testicular descent and maldescent. *Annals of the Royal College of Surgeons of England*.

